# Nutritional Value of Eggplant Cultivars and Association with Sequence Variation in Genes Coding for Major Phenolics

**DOI:** 10.3390/plants11172267

**Published:** 2022-08-31

**Authors:** Vasileia Chioti, Konstantina Zeliou, Aikaterini Bakogianni, Charikleia Papaioannou, Antonis Biskinis, Constantinos Petropoulos, Fotini N. Lamari, Vasileios Papasotiropoulos

**Affiliations:** 1 Department of Agriculture, University of Patras, Nea Ktiria, 30200 Messolonghi, Greece; 2 Laboratory of Genetics, Department of Biology, University of Patras, 26504 Patras, Greece; 3 Laboratory of Pharmacognosy and Chemistry of Natural Products, Department of Pharmacy, University of Patras, 26504 Patras, Greece; 4 Division of Statistics, Probability and Operational Research, Department of Mathematics, University of Patras, 26504 Patras, Greece

**Keywords:** *Solanum melongena*, phenolics, chlorogenic acid, antioxidant capacity, flavonoids, anthocyanins, cultivars, sequence variation, SNPs

## Abstract

Eggplant is a widely consumed vegetable, with significant nutritional value and high antioxidant content, mainly due to its phenolic constituents. Our goal was to determine the levels of carbohydrates, proteins, total phenolics, anthocyanins, flavonoids, chlorogenic acid, and the antioxidant capacity in thirteen eggplant cultivars cultivated in Greece and to identify sequence polymorphisms in key regulating genes of the phenylpropanoid pathway (*C4H*, *HCT*, *HQT*, *C3H*, *F3H*, *ANS*, *MYB1*), which might relate to the phytochemical content of those cultivars. The carbohydrates’ content differs among and within cultivars, while the rest of the phytochemicals differ only among cultivars. The cultivars ‘EMI’ and ’Lagkada’ scored higher than the rest in phenolics, anthocyanins, ascorbic acid, caffeoylquinic acid, and antioxidant capacity. Moreover, significant correlations were observed between various ingredients and the antioxidant capacity (FRAP and DPPH). Sequence analysis revealed several SNPs in *C4H*, *HQT*, *F3H*, *ANS*, and *MYB1* among the cultivars studied. According to chi-square and logistic regression analyses, the missense mutation *C4H*4-108 correlates significantly with flavonoids, anthocyanins, and proteins; the synonymous mutation *HQT*-105 correlates with anthocyanins and ascorbic acid; the missense mutation *HQT*-438 correlates with flavonoids and chlorogenic acid, while the missense mutation *ANS*1-65 correlates with anthocyanins and sugars. These polymorphisms can be potentially utilized as molecular markers in eggplant breeding, while our data also contribute to the study of eggplant’s secondary metabolism and antioxidant properties.

## 1. Introduction

Eggplant (*Solanum melongena* L.) is the fifth most popular vegetable worldwide, with around 56.6 million tons (Mt) of global production. China (36.6 Mt) is the top producer, followed by India (12.7 Mt) and Egypt (1.3 Mt) (FAOSTAT, 2020, accessed on 4 May 2022). The eggplant was introduced in Greece, around the 13th century as an ornamental plant, but now is an important part of the Greek gastronomy and the Mediterranean diet, with many local landraces possessing considerable morphological and genetic diversity [1].

Eggplant is a low-calorie vegetable with high nutritional value, due to the presence of a wide array of elements such as fibers, proteins, phenolics, vitamins, minerals, etc. [2,3]. It is also considered among the top vegetables with high antioxidant activity against peroxyl and hydroxyl radicals and no pro-oxidant activity [4]. The predominant phenolic (70–90%) in the eggplant flesh is chlorogenic acid (5-*O*-caffeoylquinic acid), while other caffeoylquinic acid isomers/derivatives and flavonol (quercetin and myricetin) glycosides are present in low quantities [5,6]. Anthocyanins are mainly located in the fruit’s peel and are responsible for the usual deep purple color [7]. Many of those phenolic compounds have antioxidant activities, conferring high nutritional value and health-promoting effects on this vegetable [8,9]. The quantity and composition of phenolics depend highly on the cultivar [5,10,11], season [12], and cultivation technique [13]. Conventional breeding techniques, based on selection and hybridization, have shown great potential for enhancing the content of bioactive compounds in a wide range of plants [14]. An increasing number of breeding programs, aiming at improving the phenolic content, have started so far, including the use of eggplant wild relatives from the primary, secondary, and tertiary gene pools, which are rich in phenolics [15,16,17,18]. Increasing the chlorogenic acid content is attracting significant interest, due to its anti-inflammatory and neuroprotective activities in humans [19], while efforts to increase the anthocyanin content in eggplants have also been launched [20,21].

Recent developments regarding the genome sequence and annotation of important genes [22,23,24] have provided significant information on the biosynthesis of phenolic acids and flavonoids in eggplant. Such genomic information is used to identify novel genes and alleles having a role in the formation and accumulation of phenolics, being also of high interest for marker-assisted selection and breeding [14]. Several genes, either regulatory or structural, are involved in the chlorogenic acid and flavonoid biosynthesis in eggplant. These genes include the following: cinnamate 4-hydroxylase, (*C4H*); hydroxycinnamoyl-CoA shikimate/quinate hydroxycinnamoyl transferase, (*HCT*); p-coumarate 3-hydroxylase, (*C3H*); hydroxycinnamoyl-CoA quinate hydroxycinnamoyl transferase, (*HQT*); flavanone 3-hydroxylase (*F3H*); anthocyanidin synthase (*ANS*); and transcription factors of the R2R3-MYB family [21,24,25]. Although this gene network is generally conserved, mutations and differences in the allelic composition may be associated with metabolic changes, resulting in alterations in the composition and accumulation of those phenolics [7,23,26].

The main objective of this work was to evaluate the nutritional value of the fresh fruits of commercial and local eggplant cultivars cultivated in Greece by determining the total sugars and protein content, as well as the content of total polyphenols, flavonoids, anthocyanins, chlorogenic acid, and their antioxidant capacity. Moreover, we aimed to identify nucleotide polymorphisms in genes coding for enzymes participating in the biosynthetic pathway of phenolics and flavonoids and investigate if they associate with variations in the chemical content of the eggplant cultivars, thus highlighting their possible utility as molecular markers in breeding schemes for improving those traits.

## 2. Results–Discussion

### 2.1. Fruit Quality Characteristics

The average fresh fruit weight for each cultivar is presented in Table 1; ‘EMI’ had the heaviest fruits, while ‘NiloF1’ had those that were less heavy.

The carbohydrate and protein content of each cultivar are summarized in Table S1. Carbohydrates differ among and within cultivars; in ‘Monarca F1’ their content is approximately three- to four-fold higher than ‘Angela F1’ and ‘Lato F1’; however, it also varies within ‘Lagkada’ and ‘Leticia F1’ (standard deviation and standard error values >10%). Differentiation in protein content is observed only among cultivars, thus ‘Monarca F1’, ‘Tsakoniki’, and ‘Cristal F1’ have values between 330 and 366 mg per 100 g of fresh weight, while ‘Lato F1’, ‘Blanchette F1’, ‘Sabelle F1’, and ‘Samantha F1’ have lower content (between 113 and 155 mg per 100 g of fresh weight).

In Table S2, the total content of phenolics, monomeric anthocyanins, flavonoids, and caffeoylquinic acid (expressed per 100 g of fresh eggplant fruit), are presented. Total phenols and total flavonoids were estimated by colorimetric assays, but since these methods are not so specific, we performed HPLC analysis too. The two major peaks detected correspond to chlorogenic acid isomers (68–87% of total peak area at 325 nm) (Figure S1), in accordance with previous studies [3,5,6]; their total peak area was used for the determination of total caffeoylquinic acid with an external standard. Total phenolic content differs significantly among cultivars; ‘Lagkada’ has the highest concentration of phenolics, followed by ‘Nilo F1’ and ‘EMI’, while ‘Lato F1’ and ‘Angela F1’ have the lowest. Total monomeric anthocyanins are very low (≤ 1 mg per 100 g of fresh fruit) in cultivars whose pericarp lacks purple color (‘Lato F1’, ‘Blanchette F1’, and ‘Samantha F1’) (see Table 1). On the other hand, the anthocyanins’ concentration is higher (8.5 to 10.4 per 100 g of fresh fruit) in the deep purple cultivars (‘EMI’, ‘Lagkada’, ‘Leticia F1’, ‘Monarca F1’, ‘Cristal F1’), except for ‘Nilo F1’. In purple and striped cultivars, the anthocyanins’ concentration is intermediate. Total flavonoids are below 65 mg per 100 g of fresh fruit in ‘Angela F1’, ‘EMI’, ‘Monarca F1’, ‘Blanchette F1’, and ‘Tsakoniki’, and above 100 mg (up to 137 mg) in ‘Lagkada’, ‘Leticia F1’, ‘Samantha F1’, and ‘Cristal F1’. With regards to chlorogenic acid, its amount is higher than 43.0 mg per 100 g of fresh fruit in ‘EMI’, ‘Lagkada’, ‘Nilo F1’, and ‘Cristal F1’, while it is lower than 30.0 mg in ‘Sabelle F1’and ‘Samantha F1’.

In Table S3, the antioxidant capacity of eggplant fruit extracts is presented. ‘Nilo F1’ has the highest FRAP values followed by ‘Lagkada’ and ‘EMI’. ‘EMI’ and ‘Lagkada’ have higher DPPH values than the rest, while ‘Blanchette F1’ has the lowest.

**Figure 1:**
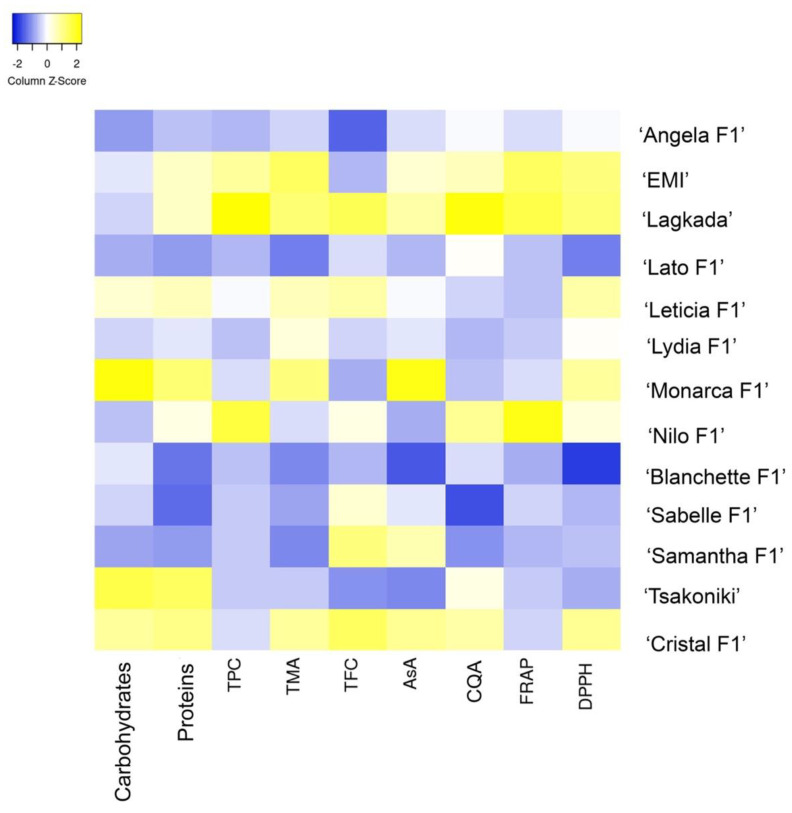
Heat map showing the variation of total sugars, proteins, phenolics (TPC), anthocyanins (TMA), flavonoids (TFC), ascorbic acid (AsA), caffeoylquinic acid (CQA), and the antioxidant properties by FRAP and DPPH scavenging assays in 13 eggplant cultivars. Standardized values (z-scores) of mean values are depicted with a color scale (from blue to yellow).

A heatmap of the fruit quality characteristics demonstrates their main differences in Figure 1, whereas the data are presented in Tables S1–S3.

Compared to the rest, ‘EMI’ and ‘Lagkada’ scored higher in phenolics, anthocyanins, ascorbic acid, caffeoylquinic acid, and antioxidant capacity (Figure 1). This fact along with the high fruit weight of ‘EMI’ highlight the nutritional importance of these cultivars and the need to preserve them. Each of the other hybrids has its own merits; ‘Monarca F1’, ‘Nilo F1’, and ‘Cristal F1’ stand out for their high antioxidant content.

Correlations between fruit weight and different chemical ingredients are presented in Table 2. Fruit weight is weakly correlated with total phenolics and anthocyanins, and moderately with ascorbic acid. The protein content is weakly correlated with sugars and moderately with total phenolics, anthocyanins, and ascorbic acid; similarly, the sugars’ content is weakly correlated with the above compounds. A higher but still moderate correlation (r = 0.689) was observed between total phenolic content and anthocyanins, whereas the correlation of the flavonoid content with total phenolics was lower (r = 0.449). No correlation was observed between flavonoids and anthocyanins whilst anthocyanins were moderately correlated with ascorbic acid (r = 0.689). Moreover, caffeoylquinic acid content weakly correlates with phenolics, flavonoids, and anthocyanins. It should be noted that the colorimetric estimation of total phenolic content with Folin-Ciocalteu is considered a non-specific assay which reflects more accurately the reducing capacity of antioxidants. Thus, the strongest correlations (0.7 < r < 0.9) were observed between DPPH and anthocyanins, phenolics, and ascorbic acid, confirming that these metabolites are capable of radical scavenging [11,27,28]. Significant correlations of FRAP with total phenolics, anthocyanins, ascorbic, and caffeoylquinic acid were also observed, while FRAP and DPPH are weakly correlated. Since the Folin-Ciocalteu assay, FRAP and DPPH are all electron-based assays measuring the reducing capacity, the different values of their correlations (generally weak to medium) reflect differences in their working principles; FRAP and Folin-Ciocalteu measure reducing capacity in aqueous media at low and high pH, respectively, whereas DPPH assay measures the radical scavenging in a methanolic medium which dissolves better lipophilic compounds.

### 2.2. Exon Sequences and Translation

In this study, exons of six major genes of the phenylpropanoid pathway (*C4H*, *HCT*, *HQT*, *C3H*, *F3H*, *ANS*), along with the transcription factor MYB1, were analyzed by sequencing, and the nucleotide polymorphisms revealed are shown in Table 3 and Figure S2.

Following sequence alignment and SNP detection, we attempted to examine possible associations between those SNPs with some of the metabolites mentioned before. To do so, initially, we performed a chi-square (X^2^) test, and SNPs that correlated with the phytochemicals were further investigated by logistic regression. These results are presented in Table 4. A schematic representation of the key biosynthetic steps leading to the formation of CQAs, flavonoids, and anthocyanins in eggplants is shown in Figure S3.

### 2.3. Gene Mutations, SNPs, and Metabolites Associations

Hydroxycinnamoyl-CoA shikimate/quinate hydroxycinnamoyl transferase’s (*HCT*) coding region is divided into three exons of which the third (*HCT*3) was selected. No polymorphism was detected in the cultivars screened. Moreover, p-coumarate 3-hydroxylase’s (*C3H*) coding region is also divided into three exons (*C3H*1, *C3H*2, and *C3H*3), which were all included in our study; likewise, no polymorphism was detected among the different genotypes.

In flavanone 3-hydroxylase (*F3H*), two SNPs were detected in two (*F3H*1 and *F3H*2) out of the three exons studied. At position 163, four out of five ‘Lato F1’ plants had a double peak of adenine/guanine, while one plant of this cultivar, as well as the plants of the remaining cultivars including the reference in Kazusa Eggplant Genome DataBase, have guanine. The triplet containing adenine translates into lysine, while that with guanine translates into glutamic acid. Nevertheless, no correlation was found with any of the phytochemicals studied.

*MYB1* has three exons and we analyzed all of them. Only one ‘Monarca F1’plant had a different SNP at alignment position 52 with a double peak of A/T while all the rest have T. It is a missense mutation that translates to tryptophan when there is adenine and to arginine in the presence of thymine. Meanwhile, by comparing our sequences with the reference sequence we noticed a missense mutation (position 737) and three synonymous (positions 675, 678, and 760). The SNPs that were found in TF MYB1 were not studied further because they were detected only in comparison to the reference sequence, while the missense mutation that was found occurred only in one plant from a single cultivar.

Anthocyanidin synthase’s (*ANS*) coding region is divided into two exons and we studied the first one (*ANS*1). In *ANS*1, we detected four substitutions (two missense mutations at positions 65 and 141 and two synonymous at positions 186 and 272), a finding that was also observed by Chen et al. [29] for the same gene. Only the SNP at position 65 had a significant correlation with anthocyanins and sugars, showing that homozygotes tend to have a high content of both compounds. Other studies [25,30,31] have shown that the level of transcription of late biosynthetic genes such as *ANS* correlates with anthocyanins. The correlation of the *ANS*1 polymorphism at position 65 with sugars seems reasonable by considering the weak correlation of sugars to the total phenolics and anthocyanins recorded before and might be attributed to the link of shikimate and carbohydrate metabolic pathways.

Cinnamate 4-hydroxylase’s coding region consists of four exons from which *C4H*2 and *C4H*4 were selected. We observed two polymorphisms in *C4H*4: a synonymous mutation at position 413 which did not show any correlations with the phytochemical data; and a missense (A>G) mutation at position 108 resulting in Thr when there was adenine and Ala in the case of guanine, which correlates with flavonoids, anthocyanins, and proteins. Schilmiller et al. [32] characterized different allelic series of *Arabidopsis ref 3* mutants harboring a missense mutation in *C4H* and they found that these mutations affect protein stability and/or reduced enzyme function.

We studied both exons of hydroxycinnamoyl quinate hydroxycinnamoyl transferase’s (*HQT*) coding region. We discovered four substitutions: two synonymous (positions 105 and 996) and two missenses (positions 438 and 565). According to our analysis, those at positions 105 and 438 are statistically significant. *HQT*-105 correlates with anthocyanins and ascorbic acid (AsA) while *HQT*-438 with flavonoids and chlorogenic acid (CQA).

A correlation between HQT and chlorogenic acid was expected since HQT is an enzyme used in many steps of CQA’s biosynthetic pathway. Moreover, in their study, Hettiarachi and Hettiarachchi [33] demonstrated that overexpression of *HQT* cDNA clones in tomatoes and tobacco leads to a higher concentration of CQA. The rest of the correlations are not so explicit, because flavonoids and anthocyanins are different end products (branched pathways) than CQA and ascorbic acid. In Table 2, the weak correlations of CQA with TFC and TMA, and the moderate ones of AsA with TMA may be attributed to many factors and not necessarily to the specific mutation. In their study, Page et al. (2012) [34] demonstrated that ascorbate is a positive regulator of anthocyanin biosynthesis in *Arabidopsis* and that the transcripts of the genes controlling flavonoid and anthocyanin biosynthesis (except for *C4H*) were also increased during high light acclimation. Concerning the significant correlations of *HQT*-105 with anthocyanins, we can assume that there is a link between HQT and CHS; 4-coumaroyl-CoA is a biosynthetic precursor of caffeoyl-CoA that esterifies with quinic acid by HQT to produce CQA and, at the same time, is a substrate of CHS to produce naringenin chalcone with which the production of flavonoids (and then anthocyanins) begins [25]. In this manner, the correlations between CQA and anthocyanins and *HQT*-105 with anthocyanins can be explained.

## 3. Materials and Methods

### 3.1. Plant Material

For this study, three local and ten commercial eggplant cultivars, widely cultivated in Greece were selected; the origin, cultivar name, as well as fruit characteristics (shape and color) are presented in Table 1. Seeds and mature fruits of the commercial cultivars were obtained from the Agricultural Cooperatives "Anatoli" and ‘’To Nisi’’ (Ierapetra, Greece), as well as Rijk Zwaan (De Lier, The Netherlands) and Semillas Fitó (Barcelona, Spain), whereas for the three Greek local cultivars, by the Institute of Plant Breeding and Genetic Resources, Hellenic Agricultural Organization (Thermi, Greece) and the Agricultural Cooperative of Leonidio (Leonidio, Greece). At least three fruits per cultivar (from different plants) at commercial maturity were used for the chemical characterization. Fresh and young leaves from three to five plants per accession were collected for DNA extraction.

### 3.2. Chemical Analysis

#### 3.2.1. Fruit Extraction

Each vegetable at commercial maturity was thoroughly washed with de-ionized water, drained, patted dry with paper towels, had its calyx removed, cut in half (vertical section-from tip to base), and then in quarters by cross-section. One of the quarters was weighed and homogenized in a home electric chopper machine for ten seconds, while an extraction solution of methanol, distilled water, and formic acid (analytical grade, Sigma-Aldrich, Saint Louis, MO, USA) (48.5/50.0/1.5, *v*/*v*/*v*) was added. The mixture was transferred into a borosilicate glass beaker and was left for 1 h at magnetic stirring. Finally, the mixture was filtered through a double paper filter, the extract was collected and kept at −20 °C until use. A fraction was lyophilized to dry (Labconco FreeZone 6, Kansas City, MO, USA).

#### 3.2.2. Colorimetric Determination of Total Phenolics, Total Monomeric Anthocyanins, Total Flavonoids, Proteins, and Sugars

All determinations were performed in the aqueous methanolic extracts and the absorbance was measured in a UV/vis microplate reader (Sunrise, Tecan Group Ltd, Männedorf, Switzerland) against blanks. Each sample was tested in triplicates.

Proteins were estimated according to the Bradford method (1976) [35]. In detail, 5 μL of each sample extract (120 mg mL^−1^ fresh eggplant weight) were added to 250 μL of Bradford reagent (AppliChem, Darmstadt, Germany). The plate was kept in the dark for 45 min at 37 °C, after which the absorbance was read at 595 nm. The protein content was expressed as mg of bovine serum albumin (BSA) equivalents per 100 g fresh weight (FW), according to the calibration curve *y* = 0.192·*x*+0.003, R² = 0.990·(0.05, 0.25, 0.30, 0.40, 0.50, 0.80, 1.00, and 1.20 mg mL^−1^ BSA).

Total sugars were measured with the anthrone-sulfuric acid method adapted for 96 well plates [36]. Briefly, 100 μL anthrone solution (freshly prepared) was added to 40 μL of the sample (20.0 mg mL^−1^ FW). The absorbance was read at 620 nm. Total carbohydrate content was expressed as mg glucose equivalents per 100 g FW, according to the calibration curve *y* = 4.520·*x*+0.052, R² = 0.999·(0.031, 0.062, 0.125, 0.250 and 0.500 mg mL^−1^ glucose).

Total phenolics were estimated according to the Folin-Ciocalteu reagent method [37] with minor modifications. In brief, 4 μL of each sample extract (20 mg mL^−1^ F.W.) were added to 80 μL of NaCO_3_ (7.5%, w/v), and then 100 μL of Folin–Ciocalteu reagent (Sigma-Aldrich, Saint Louis, MO, USA) was added. Absorbance was read at 620 nm and the total polyphenolic content was expressed as mg of gallic acid equivalents (GAE) per 100 g FW according to the equation *y* = 0.986·*x* + 0.237, R² = 0.997·(0.12, 0.25, 0.50, 1.00, and 2.00 mg·mL^−1^ gallic acid).

The estimation of total flavonoids was based on the Woisky and Salatino (1998) [38] method. In detail, 16 μL of aqueous sample extract (20 mg mL^−1^ dry extract weight) were added to a mixture containing 40 μL of ethanol (95%), 75 μL of water, and 5 μL aqueous potassium acetate (1 M) solution (CH_3_COOK, >99% AppliChem GmbH, Darmstadt, Germany). Then, 5 μL of aqueous aluminum chloride (10% *w*/*v*) solution (AlCl _3_ hexahydrate, >99% reagent plus grade, Sigma-Aldrich, Saint Louis, MO, USA) was added, and the mixture was thoroughly mixed, and the absorbance was read at 405 nm after 45 min. The total flavonoid content was expressed as mg of quercetin equivalents per 100 g FW, using the standard curve *y* = 0.951·*x* + 0.097, R ² = 0.981 (0.12, 0.25, 0.50, and 1.00 mg·mL^−1^ of quercetin).

Total monomeric anthocyanins (TMA) were estimated with the pH differential method [39,40]. Absorbance was read at 620 nm (λ_min_) and at 520 nm (λ_max_) and the results were calculated with the equation TMA (cyanidin-3-glucoside equivalents mg/L) = (A × MW ×DF × 10^3^) / (ε × 1), where A= (A_520_ – A _620_) pH 1.0 – (A_520_ – A _620_) pH 4.5; M.W. (molecular weight) = 449.2 g/mol; DF = dilution factor; 1 = path length in cm; ε = 26,900 L cm^−1^ mol^−1^, molar extinction coefficient for cyanidin 3-*O*-β-D-glucoside. 10^3^: factor to convert g to mg.

#### 3.2.3. Quantification of Chlorogenic Acid Content by Using an HPLC-UV Method

The HPLC analysis was performed on an HPLC–DAD Dionex Ultimate 3000 system (Thermo Fisher Scientific, Waltham, MA, USA) consisting of a pump (LPG–3400 A), a thermostated column compartment (TCC–3100), and a Photodiode Array Detector (DAD−3000). Separation was performed on a C18 column (Luna C18; 250 × 4.6 mm, 5 µm; Phenomenex, Torrance, CA, USA) with a coupled guard column cartridge C18 (4.0 × 3.0 mm, 5 µm; Phenomenex, Torrance, CA, USA) set at 30 °C. The injection volume was 20 μL and the concentration of the extract was 150 mg of fresh weight per mL. The elution system consisted of ammonium acetate (pH 4.5; 10 mM), and acetonitrile in a combination of gradient and isocratic steps. In detail, initial elution was performed with 85:15, *v*/*v* for 10 min. Then, the acetonitrile content was raised to 100:0, *v*/*v*, in 8 min, and kept constant for 2 min up to 85:15, *v*/*v*, in 2 min and kept constant for 5 min. The mobile phase returned to the initial 85:15, *v*/*v* in 2 min, and kept constant for 3 min. The flow rate of the mobile phase was 1 mL min^−1^. Data were collected at 325 nm, stored, and integrated with Chromeleon v 6.80 Systems software. Chlorogenic acid was identified by its retention time and its UV/vis spectrum against a reference standard (5–*O*–caffeoylquinic acid, ≥99%) purchased from Extrasynthese (Genay, France). Quantification was performed by the external standard calibration method with the equation *y* = 2.162·*x* + 11.370 (R^2^ = 0.992) (10.0, 20.0, 50.0, 100.0, 150.0 and 200.0 μg mL^−1^ chlorogenic acid).

#### 3.2.4. Colorimetric Determination of Antioxidant Capacity (DPPH and FRAP)

The antioxidant activity was determined with the 2,2-diphenyl-1-picrylhydrazyl (DPPH) method [41,42]. For the DPPH method, 5 μL of extracts at 225 mg mL^−1^ FW were mixed thoroughly with 195 μL of methanolic DPPH (0.1 mM) in 96 well plates. After a 30 min incubation period in the dark at room temperature, the absorbance was measured at 540 nm. The scavenging activity of samples was expressed as the percentage of inhibition as [(A_Control_-A_Sample_)/A_Control_] × 100.

In addition, we used the ferric reducing antioxidant power (FRAP) method [43], which measures the ability of antioxidants to reduce [Fe(TPTZ)_2_]^3+^ to [Fe(TPTZ)_2_]^2+^ and the results were expressed as μmol Fe^2+^ per 100 g FW. In brief, 80 μL of FRAP reagent (10 mM TPTZ solution in 40 mM HCl and 20 mM FeCl_3_·6H_2_O solution in 300 mM acetate buffer, pH 3.6) were mixed with 55 μL acetate buffer (300 mM, pH 3.6) and 60 μL of samples (20.0 mg of FW of extract mL^−1^). Absorbance was measured at 595 nm. Ferrous sulfate heptahydrate (FeSO_4_·7H_2_O, >99%, Sigma-Aldrich, Saint Louis, MO, USA) was used for the construction of a standard curve (*y* = 4.115·*x* + 0.005, *R²* = 0.995·(0.10, 0.20, 0.30, 0.04, and 0.05 μmol mL^−1^).

### 3.3. Molecular Analysis

#### 3.3.1. DNA Extraction and PCR Amplification

Total genomic DNA was extracted by homogenizing 100 mg of young leaf tissue from each accession with liquid nitrogen and following the CTAB protocol [44], with modifications. Genes coding for major enzymes of the phenolic pathway were selected for PCR amplification. Specific primer pairs for selected exons of each gene were designed based on the coding sequences deposited in Kazusa Eggplant Genome DataBase (http://eggplant.kazusa.or.jp/, accessed on 17 October 2020) [22]. Primers were designed using the NCBI primer designing tool (https://www.ncbi.nlm.nih.gov/tools/primer-blast/, accessed on 22 January 2019) and the Oligo Analysis Tool (https://eurofinsgenomics.eu/en/ecom/tools/oligo-analysis/, accessed on 22 January 2019). The exons selected, as well as the primer pair sequences, can be found as supplementary data (Tables S4 and S5, respectively).

PCR reactions were performed in a total volume of 30 μL consisting of PCR-grade water, 1Χ Taq Buffer, 0.2 mM dNTPs, 2 μM of each primer, 1 U Taq Polymerase (5 U/μL KAPA Taq DNA Polymerase), 1.5 mM MgCl_2_, and approximately 1 ng of DNA template. PCR was performed in a C1000 Touch^™^ Thermal Cycler (Bio-Rad), Hercules, CA, USA, using different annealing temperatures, depending on the gene amplified (Table S6).

For PCR products’ verification, 5 μL of each PCR product were electrophoresed in 1% agarose gels, along with a 100 bp NEB DNA ladder (New England BioLabs Inc., Ipswich, MA, USA). Amplified products were purified using the Nucleospin Gel and PCR clean-up kit (Macherey – Nagel, Düren, Germany); concentration and quality of PCR products were determined using Quawell UV-Vis Q5000 spectrophotometer (Quawell Ltd., San Jose, CA, USA) and prepared for sequencing. The gene sequences obtained were deposited in GenBank under accession numbers OP295851-OP296250: OP295851-OP295900 for *HCT*3, OP295901-OP295950 for *HQT*, OP295951-296000 for *C4H*2, OP296001-OP296050 for *C4H*4, OP296051-OP296100 for *C3H*, OP296101-OP296150 for *F3H*, OP296151-OP296200 for *MYB1*, OP296201-OP296250 for *ANS*1.

#### 3.3.2. Molecular Data Analysis

Exon sequences were manually edited using Finch TV version 1.4.0. (Geospiza Inc., Seattle, WA, USA) and SeqMan II software (DNASTAR Inc., Madison, WI, USA). For the enzymes that the whole coding sequence was selected, exon sequences were combined and analyzed as one data set. Multiple sequence alignments were performed with Sequencer Demo 4.8 (GeneCodes Co, Ann Arbor, MI, USA) and Clustal Omega multiple sequence alignment package [45,46], using the default parameters. Following multiple alignments, the nucleotide sequences from each selected genotype were compared to each other, as well as with those deposited in the Kazusa Institute database. MEGA version 7.0.26 [47] and Sequencer Demo 4.8 were used to check for the presence of Single Nucleotide Polymorphisms (SNPs). Afterward, the exon sequences were translated into protein sequences using MEGA v.7.0.26 and ExPASy translation tool (https://web.expasy.org/translate/, accessed on 23 August 2022) and standard genetic code.

### 3.4. Statistical Analysis

Means, standard deviation, and standard error for instrumental measurements were calculated using three replicates from each eggplant variety measurement. Fruits from each variety were subjected to mean comparison by one-way analysis of variance (ANOVA) using Tukey’s test at a 5% level of significance (*𝛼* = 0.05). Spearman’s correlation was performed for all variable pairs at a 5% level of significance (*𝛼* = 0.05) and estimated values of *r* > 0.90, *r* > 0.70, and *r* > 0.50 are interpreted as very high, high, and moderate, respectively. Heatmapping of the average values of the quality traits for the selected genotypes was performed with the online web tool Heatmapper [48].

Furthermore, we proceeded to find out if any significant correlation exists between the phytochemicals with molecular data, and we tested if certain SNPs (missense and synonymous) are linked with the concentration levels of total phenolics, flavonoids, anthocyanins, chlorogenic acid, ascorbic acid, proteins, and sugars. For this purpose, the concentrations of each phytochemical were divided into three groups (low, moderate, and high) by calculating their range and appointing two cut points. Cut point 1 ((range/3) + minimum value) indicates the point that divides low from moderate values and cut point 2 ((2*range/3) + minimum value) indicates the point separating moderate from high values. Then a chi-square test (X^2^) was performed to reveal the statistically important correlations. The SNPs significantly correlated were used as independent variables for the logistic regression performed afterward. This way, we tried to examine if the chemical content (dependent variables) correlates with specific mutations (SNPs). Statistical analysis was performed using IBM SPSS Statistics v.27 software.

## 4. Conclusions

*Solanum melongena* L. is one of the main edible species worldwide with high nutritional value due to its plethora of chemical constituents, that are beneficial for human health. Despite its importance, it lacks genetic and genomic tools, compared to other members of the Solanaceae family such as tomato, but recently serious efforts have been made to bridge this gap [1,23]. This study aimed to evaluate the nutritional value and antioxidant capacity of 13 eggplant cultivars cultivated in Greece, to identify mutations existing in genes coding for enzymes that play a key role in the production of flavonoids, anthocyanins, and chlorogenic acid, to investigate possible associations of those polymorphisms with the chemical composition of each cultivar and finally to evaluate if those polymorphisms could be used as molecular markers in eggplant breeding schemes.

Differences were observed within and among cultivars both at chemical and molecular levels. Cultivars ‘EMI’ and ‘Lagkada’ scored higher than the rest in phenolics, anthocyanins, ascorbic acid, caffeoylquinic acid, and antioxidant capacity. At the molecular level, out of the sixteen SNPs detected, only SNP *ANS*1*-*65 shows potential to be used as a molecular marker since homozygotes tend to have a high content of anthocyanins and sugars, but further study needs to be done. Other SNPs with potential interest are *C4H4-*108, *HQT-*105, and *HQT*-438. Breeding programs increasingly consider the concentration and content of bioactive compounds as a major breeding objective [49]. Therefore, understanding the metabolic constitution of plants and their genetic background is a prerequisite to developing strategies for improving crop compositional quality. With our study, we elucidate different aspects of the phenylpropanoid pathway and its molecular formation and identify potential targets for future studies.

## Figures and Tables

**Table 1:** Eggplant cultivars that were studied; origin and fruit’s color and shape of each cultivar; average fresh fruit weight and standard deviation. Values in parentheses represent the standard error (SE) of the measurements; n: the number of biological samples.

Cultivar	Origin	Fruit Color	Shape	Fruit Weight (g)
‘Lagkada’ (n = 3)	GR	Deep purple	Cylindrical	278.3 ± 39.6 (22.8)
‘Tsakoniki’ (n = 3)	GR	Purple with white stripes	Cylindrical	194.3 ± 19.1 (11.1)
‘ΕΜΙ’ (n = 3)	GR	Deep purple	Oval	481.7 ± 51.3 (29.6)
‘Cristal F1’ (n = 6)	ES	Deep purple	Oval	309.2 ± 31.9 (10.6)
‘Sabelle F1’ (n = 3)	NL	Light purple	Round	349.7 ± 58.4 (33.7)
‘Angela F1’ (n = 3)	NL	Purple with white stripes	Oval	236.7 ± 2.1 (1.2)
‘Nilo F1’ (n = 3)	NL	Deep purple	Cylindrical	122.7 ± 6.7 (3.8)
‘Leticia F1’ (n = 9)	NL	Deep purple	Oval	332.5 ± 48.7 (16.2)
‘Monarca F1’ (n = 3)	NL	Deep purple	Oval	262.7 ± 22.7 (13.1)
‘Lydia’ F1’ (n = 9)	NL	Purple with white stripes	Oval	270.2 ± 36.7 (15.0)
‘Samantha F1 (n = 3)	NL	Light green	Oval	304.7 ± 70.4 (40.7)
‘Lato F1’ (n = 6)	NL	White	Oval	254.7 ± 10.4 (4.2)
‘Blanchette F1’ (n = 3)	NL	White	Round	196.7 ± 17.6 (10.1)

Abbreviations: GR: Greece, NL: The Netherlands, ES: Spain.

**Table 2:** Correlation coefficients and levels of significance among the physicochemical parameters studied.

	Weight	TPC	TFC	TMA	AsA	Proteins	Sugars	CQA	FRAP	DPPH
**Weight**	-									
**TPC**	0.266 *	-								
**TFC**	0.097	0.448 ***	-							
**TMA**	0.456 ***	0.689 ***	0.167	-						
**AsA**	0.508 ***	0.502 ***	0.393 **	0.690 ***	-					
**Proteins**	0.231	0.514 ***	-0.180	0.619 ***	0.405 **	-				
**Sugars**	0.002	0.367 **	0.128	0.423 **	0.271 *	0.392 **	-			
**CQA**	−0.156	0.327 *	0.288 *	0.321 *	0.142	0.184	0.068	-		
**FRAP**	−0.033	0.422 **	0.119	0.366 **	0.290 *	0.134	0.107	0.400 *	-	
**DPPH**	0.417 **	0.761 ***	0.337 *	0.898 ***	0.703 ***	0.560 ***	0.307 *	0.379 **	0.363 **	-

* α < 0.05, ** α < 0.01, *** α < 0.001.

**Table 3:** Observed nucleotide polymorphisms in the genes studied. Those in bold represent polymorphisms observed only in comparison with the reference sequence deposited in Kazusa Eggplant Genome DataBase. Mutation types, nucleotide substitutions, and amino acid changes are also presented.

Gene/Exon	Alignment Position	Type of Mutation	Substitution	Translation
*C4H*4	108	missense	A>G	Thr>Ala
	413	synonymous	T>C	Ile
*HQT*	105	synonymous	T>C	His
	438	missense	T>G	His>Gln
	565	missense	A>G	Thr>Ala
	996	synonymous	T>C	Ser
*F3H*	163	missense	A>G	Lys>Glu
*ANS*1	65	missense	T>C	Val>Ala
	141	missense	T>A	Asp>Glu
	186	synonymous	A>G	Ser
	282	synonymous	T>C	His
*MYB1*	52	missense	T>A	Trp>Arg
	**675**	synonymous	C>A	Glu
	**678**	synonymous	C>A	Ala
	**737**	missense	T>C	Phe>Ser
	**760**	synonymous	T>C	Leu

**Table 4:** X^2^ contingency table. In the first row, enzyme names along with the observed SNP’s positions (second row) are shown. The left column shows the phytochemicals that were examined for correlation with the above-mentioned SNPs. The columns in grey represent SNPs leading to synonymous mutations, while the rest to missense mutations. The numbers in bold show the statistically significant correlations (*p* < 0.05), while those in italics have *p*-values close to 0.05. Underlined numbers represent significant correlation values also observed in the logistic regression.

	*C4H*4		*HQT*				*F3H*	*ANS*1			
	108	413	105	438	565	996	163	65	141	186	282
**TPC**	0.109	0.109	***0*** ***.0*** ***60***	0.460	0.460	0.460	0.460	0.143	0.143	0.143	0.143
**TFC**	**0** **.** **022**	**0.022**	0.877	**0.004**	**0.004**	**0.004**	***0.064***	0.000	0.000	0.000	0.000
**TMA**	**0** **.** **000**	**0.000**	**0.000**	**0.050**	**0.050**	**0.050**	0.050	**0.001**	0.001	0.001	0.001
**AsA**	0.087	0.087	**0.029**	0.130	0.130	0.130	0.068	0.098	0.098	0.098	0.098
**Proteins**	**0** **.** **027**	**0.027**	**0.000**	**0.050**	**0.050**	**0.050**	**0.050**	**0.014**	**0.014**	**0.014**	**0.014**
**Sugars**	0.145	0.145	0.077	0.494	0.494	0.494	0.494	**0.041**	**0.041**	**0.041**	**0.041**
**CQA**	0.632	0.632	0.183	**0.008**	**0.008**	**0.008**	0.347	0.526	0.526	0.526	0.526

## Data Availability

Not applicable.

## Supplementary Materials

The following supporting information can be downloaded at: https://www.mdpi.com/article/10.3390/plants11172267/s1, Figure S1: Representative chromatogram of eggplant extracts at 270 nm (upper) and at 325 nm (lower). The UV-vis spectra of the peaks at 2.83 and 3.7 min that correspond to caffeoylquinic acids are provided. Other peaks present at 270 nm were not identified; Figure S2: Sequence alignment results and SNPs identified among the eggplant cultivars; Figure S3: Schematic representation of the key biosynthetic steps leading to the formation of CQAs, flavonoids, and anthocyanins in eggplants; gene names in bold correspond to those studied in this research; Table S1: Total carbohydrates’ and proteins’ content of selected eggplant cultivars; Table S2: Total phenolics (TPC), total monomeric anthocyanins (TMA), total flavonoids (TFC), ascorbic (AsA), and caffeoylquinic acid (CQA) content of the selected eggplant cultivars; Table S3: Antioxidant capacity (FRAP and DPPH) of the selected eggplant cultivars; Table S4: Enzymes names and symbols, enzyme name used in Kazusa Eggplant Genome DataBase, total number of exons, exons selected, and their sizes; Table S5: Genes, exon number, and size, PCR primers used; Table S6: PCR conditions for each gene studied.
