# Supplementary material for: Nutritional Value of Eggplant Cultivars and Association with Sequence Variation in Genes Coding for Major Phenolics
Source: Plants (Basel). 2022 Aug 31;11(17):2267. doi: 10.3390/plants11172267 (PMC9460228; doi:10.3390/plants11172267)
Supplement: Supplementary file 1 [file plants-11-02267-s001.zip › Supplementary Table S1.pdf]

Table S1. Total carbohydrates and total protein content of selected eggplant cultivars.

**Cultivar**	**Carbohydrates** (mg of Glucose Equivalents per 100g of F.W.)	**Proteins** (mg of BSA Equivalents per 100g of F.W.)
'Angela F1' (n=3)	519.2±5.6 (3.2)	190.1±1.5 (0.9)
'EMI' (n=3)	930.1±78.4 (45.3)	282.1±13.2 (7.6)
'Lagkada' (n=3)	816.4±157.9 (91.2)	283.9±8.6 (5.0)
'Lato F1' (n=6)	634.3±33.3 (13.6)	155.0±9.9 (4.0)
'Leticia F1' (n=9)	1225.7±725.9 (242.0)	288.1±36.3 (14.8)
'Lydia F1' (n=9)	808.4±70.4 (23.5)	217.6±105.8 (43.2)
'Monarca F1' (n=3)	2150.3±189.4 (109.4)	347.5±17.3 (10.0)
'Nilo F1 (n=3)	727.2±80.8 (46.6)	258.6±7.0 (4.0)
'Blanchette F1' (n=3)	902.7±37.7 (21.7)	121.7±4.3 (2.5)
'Sabelle F1' (n=3)	805.1±34.8 (20.1)	112.8±3.4 (1.1)
'Samantha F1' (n=3)	569.1±22.1 (12.8)	154.3±3.4 (1.1)
'Tsakoniki' (n=3)	1874.7±171.6 (99.1)	366.0±11.4 (6.6)
'Cristal F1' (n=6)	1473.5±301.0 (122.9)	330.8±64.6 (26.4)
