# Supplementary material for: Nutritional Value of Eggplant Cultivars and Association with Sequence Variation in Genes Coding for Major Phenolics
Source: Plants (Basel). 2022 Aug 31;11(17):2267. doi: 10.3390/plants11172267 (PMC9460228; doi:10.3390/plants11172267)
Supplement: Supplementary file 1 [file plants-11-02267-s001.zip › Supplementary Table S2.pdf]

Table S2. Total phenolics (TPC), total monomeric anthocyanins (TMA), total flavonoids (TFC), ascorbic (AsA), and caffeoylquinic acid (CQA) content of the selected eggplant cultivars.

**Cultivar**	**TPC** (mg of Gallic Acid Equivalents per 100g of F.W.)	**TMA** (mg of Cyd-3-Glu Equivalents per 100g of F.W)	**TFC** (mg of Quercetin Equivalents per 100g of F.W)	**AsA (mg/100g F.W.)**	**CQA** (mg of 3CQA Equivalents per 100g of F.W.)
'Angela F1'(n=3)	1126.1±25.1 (14.5)	3.40±0.08 (0.05)	32.2±10.4 (6.0)	127.5±2.5 (1.4)	37.0±4.5 (2.6)
EMI (n=3)	5710.5±316.3 (182.6)	10.44±0.04 (0.02)	60.4±4.9 (3.4)	145.0±1.2 (0.7)	43.0±12.0 (6.9)
'Lagkada' (n=3)	10049.1±494.3 (285.4)	9.90±0.22 (0.13)	137.0±1.0 (0.7)	152.8±1.8 (1.0)	59.8±1.9 (1.1)
'Lato F1' (n=6)	1129.5±37.7 (15.4)	0.32±0.02 (0.01)	74.2±8.0 (3.3)	120.9±8.1 (3.3)	38.1±5.9 (2.4)
'Leticia F1' (n=9)	3099.9 ±180.5 (60.2)	7.38±2.15 (0.72)	112.1±37.6 (12.5)	134.6±3.6 (1.2)	32.6±4.3 (1.4)
'Lydia F1' (n=9)	1388.9±32.9 (11.0)	6.38±0.23 (0.08)	72.1±26.7 (8.9)	130.1±3.0 (1.0)	30.3±17.2 (5.7)
'Monarca F1' (n=3)	2164.3±104.9 (60.5)	9.55±0.14 (0.08)	59.8±2.5 (1.4)	179.9±2.3 (1.3)	30.8±8.6 (5.0)
'Nilo F1' (n=3)	8322.5±381.9 (220.5)	3.84±0.19 (0.11)	93.2±10.5 (7.4)	118.9±3.6 (2.1)	47.7±14.6 (8.4)
'Blanchette F1' (n=3)	1286.4±14.4 (8.3)	0.56±0.05 (0.03)	60.2±5.6 (3.2)	100.9±0.7 (0.4)	33.4±10.4 (6.2)
'Sabelle F1' (n=3)	1559.5±37.6 (21.7)	1.5±0.07 (0.04)	99.8±10.2 (5.9)	130.6±1.0 (0.6)	18.7±9.2 (5.3)
'Samantha F1'(n=3)	1582.9±36.1 (20.9)	0.66±0.07 (0.04)	123.6±15.3 (8.8)	150.5±1.9 (1.1)	26.4±3.1 (1.8)
'Tsakoniki' (n=3)	1739.7± 28.1 (16.2)	3.08±0.11 (0.06)	49.3±5.3 (3.1)	111.1±0.9 (0.5)	39.3±3.9 (2.2)
'Cristal' (n=6)	2146.3±123.6 (50.5)	8.46±1.68 (0.68)	135.7±44.5 (18.2)	154.9±3.4 (1.4)	45.8±9.9 (4.0)
