# Supplementary material for: Nutritional Value of Eggplant Cultivars and Association with Sequence Variation in Genes Coding for Major Phenolics
Source: Plants (Basel). 2022 Aug 31;11(17):2267. doi: 10.3390/plants11172267 (PMC9460228; doi:10.3390/plants11172267)
Supplement: Supplementary file 1 [file plants-11-02267-s001.zip › Supplementary Table S3.pdf]

**Table S3**. Antioxidant capacity (FRAP and DPPH) of the selected eggplant cultivars

| **Cultivar** | **FRAP** (μmol FeSO4 per 100g F.W.) | **DPPH** % scavenging of DPPH radical (at 22.5 g of F.W. per 100mL) |
|---|---|---|
| 'Angela F1' (n=3) | 1525.7±37.3 (21.6) | 55.8±0.4 (0.2) |
| 'EMI' (n=3) | 1985.7±36.8 (21.2) | 83.6±9.2 (5.3)) |
| 'Lagkada' (n=3) | 2023.6±25.6 (14.8) | 84.3±2.6 (1.5) |
| 'Lato F1' (n=6) | 1466.0±42.9 (17.5) | 29.3±7.3 (3.0) |
| 'Leticia F1' (n=9) | 1468.9±79.4 (26.5) | 74.2±9.9 (3.3) |
| 'Lydia F1' (n=9) | 1487.5±48.0 (16.0) | 57.9±4.6 (1.5) |
| 'Monarca F1' (n=3) | 1538.1±40.8 (23.6) | 76.5±1.8 (1.0) |
| 'Nilo F1' (n=3) | 2155.0±90.6 (52.3) | 63.4±2.6 (1.5) |
| 'Blanchette F1' (n=3) | 1413.1±72.7 (42.0) | 16.2±1.5 (0.8) |
| 'Sabelle F1' (n=3) | 1505.7±114.9 (66.3) | 42.7±2.1 (1.2) |
| 'Samantha F1' (n=3) | 1447.8±25.7(14.8) | 43.2±2.3(1.4) |
| 'Tsakoniki' (n=3) | 1475.1±33.0(19.0) | 38.9±1.7(1.0) |
| 'Cristal F1' (n=6) | 1512.4±58.4(19.5) | 78.2±6.3(2.1) |
