# Supplementary material for: Nutritional Value of Eggplant Cultivars and Association with Sequence Variation in Genes Coding for Major Phenolics
Source: Plants (Basel). 2022 Aug 31;11(17):2267. doi: 10.3390/plants11172267 (PMC9460228; doi:10.3390/plants11172267)
Supplement: Supplementary file 1 [file plants-11-02267-s001.zip › Supplementary Table S4.pdf]

Table S4: Enzymes names and symbols, enzyme name used in in Kazusa Eggplant Genome DataBase, total number of exons, exons selected, and their sizes.

Enzymes/Transcription Factor		Name in Kazusa Genome DataBase	Total Number of Exons	Selected Exons	Code Name	Size
Cinnamate 4-hydroxylase	C4H	Sme2.5_00001.1_g00048.1	4	exon 2	C4H2	722bp
				exon 4	C4H4	581bp
Hydroxycinnamoyl-CoA shikimate/quinate hydroxycinnamoyl transferase	HCT	Sme2.5_04555.1_g00001.1	3	exon 3	HCT3	689bp
Hydroxycinnamoyl quinate hydroxycinnamoyl transferase	HQT	Sme2.5_00673.1_g00011.1	2	exon 1	HQT1	1077bp
				exon 2	HQT2	
P-coumarate 3-hydroxylase	C3H	Sme2.5_00529.1_g00005.1	3	exon 1	C3H1	1546bp
				exon 2	C3H2	
				exon 3	C3H3	
Flavanone 3-hydroxylase	F3H	Sme2.5_00015.1_g00020.1	3	exon 1	F3H1	1101bp
				exon 2	F3H2	
				exon 3	F3H3	
Anthocyanidin Synthase	ANS	Sme2.5_01638.1_g00005.1	2	exon 1	ANS1	512bp
MYB1 (TF)		Sme2.5_05099.1_g00002.1	3	exons 1&2	MYB1_20	823bp
				exon 3	MYB3	
