# Supplementary material for: Nutritional Value of Eggplant Cultivars and Association with Sequence Variation in Genes Coding for Major Phenolics
Source: Plants (Basel). 2022 Aug 31;11(17):2267. doi: 10.3390/plants11172267 (PMC9460228; doi:10.3390/plants11172267)
Supplement: Supplementary file 1 [file plants-11-02267-s001.zip › Supplementary Table S5.pdf]

Table S5: Genes, exon number and size, PCR primers used.

Gene	Exon (number of bp)		Primer Sequence
C4H	2 (738bp)	F	TTACTGGAGAAGACCTTAAT
		R	GGAGTCAAAATAAACTGAAC
	4 (586bp)	F	TCTTAATCATTGTCGTGAAC
		R	TCTTCAGAACCCCTTAAC
C3H	1 (506bp)	F	GGGAATAAAGAGTAGCAATTA
		R	AAAAACCTACTACGCCTT
	2 (400bp)	F	GTATCAAAGTGAACTCAAAG
		R	ATGTCAGAATGTGGTAATTT
	3 (645bp)	F	CATGGGTAACCAATTCTTC
		R	TGAACTGATCTCCCTTAAAT
HCT	3 (736bp)	F	CGTGATCCACCTCAG
		R	AGCTTGTTTCAAATGTCATA
HQT	1 (189bp)	F	ATACCAATAACTACAACAG
		R	GAAAGAAGCATTAAGCAAT
	2 (877bp)	F	ATAAGCATTTCATTCACGA
		R	CTGATAGTGTGAAAATTCTTT
F3H	1 (360bp)	F	CTACACACAACTCCTAATC
		R	GTGACGTGACTTATCATTTT
	2 (427bp)	F	AATCAAATGGAAGAGAGTTA
		R	AACTGTCCAAAATCACATAA
	3 (311bp)	F	TGAGAAATATAAAGCAACTG
		R	AGACACTAGGGTTAAAAGA
ANS	1 (512bp)	F	ATTGAAAACTCTGGAATCAT
		R	TTGGTGTTCAGTCTATATCT
MYB1	1 & 2 (264bp)	F	ACGTTCACCAAAGGGTTTGC
		R	GTGCACCTGTTGCCTAAGAG
	3 (559bp)	F	TCTCATGTCGACAAAAAC
		R	CACTTAATATGACATGATGC
