# Supplementary material for: Nutritional Value of Eggplant Cultivars and Association with Sequence Variation in Genes Coding for Major Phenolics
Source: Plants (Basel). 2022 Aug 31;11(17):2267. doi: 10.3390/plants11172267 (PMC9460228; doi:10.3390/plants11172267)
Supplement: Supplementary file 1 [file plants-11-02267-s001.zip › Supplementary Table S6.pdf]

Table S6: PCR conditions for each gene studied.

Gene(s)	***C4H*1 και *C4H*2**		***ANS*1**		***F3H*2**		***F3H*1**		***F3H*3**	
Step 1	95˚C	5min	95˚C	5min	95˚C	5min	95˚C	5min	95˚C	5min
Step 2	95˚C	40sec	95˚C	40sec	95˚C	40sec	95˚C	40sec	95˚C	40sec
Step 3	**49˚C**	**1min**	**45.5˚C**	**1min**	**47˚C**	**45sec**	**49˚C**	**45sec**	**47˚C**	**40sec**
Step 4	72˚C	**1min 10 sec**	72˚C	**1min 10 sec**	72˚C	**1min**	72˚C	**1min**	72˚C	**1min**
Step 5	Steps 2 to 4 for 34 cycles									
Step 6	72˚C	10min	72˚C	10min	72˚C	10min	72˚C	10min	72˚C	10min
Step 7	4˚C	∞	4˚C	∞	4˚C	∞	4˚C	∞	4˚C	∞

Gene(s)	***HQT*1**		***HQT*2**		***HCT*3**		***MYB*1&2**		***MYB*3**	
Step 1	95˚C	5min	95˚C	5min	95˚C	5min	95˚C	5min	95˚C	5min
Step 2	95˚C	40sec	95˚C	40sec	95˚C	40sec	95˚C	**45sec**	95˚C	**45sec**
Step 3	**46˚C**	**1min**	**46˚C**	**30sec**	**48˚C**	**1min**	**57˚C**	**40sec**	**50˚C**	**40sec**
Step 4	72˚C	**1min 20 sec**	72˚C	**1min**	72˚C	**1min 10sec**	72˚C	**1min**	72˚C	**1min**
Step 5	Steps 2 to 4 for 34 cycles									
Step 6	72˚C	10min	72˚C	10min	72˚C	10min	72˚C	10min	72˚C	10min
Step 7	4˚C	∞	4˚C	∞	4˚C	∞	4˚C	∞	4˚C	∞
