# Supplementary figures and images for: Nutritional Value of Eggplant Cultivars and Association with Sequence Variation in Genes Coding for Major Phenolics
Source: Plants (Basel). 2022 Aug 31;11(17):2267. doi: 10.3390/plants11172267 (PMC9460228; doi:10.3390/plants11172267)

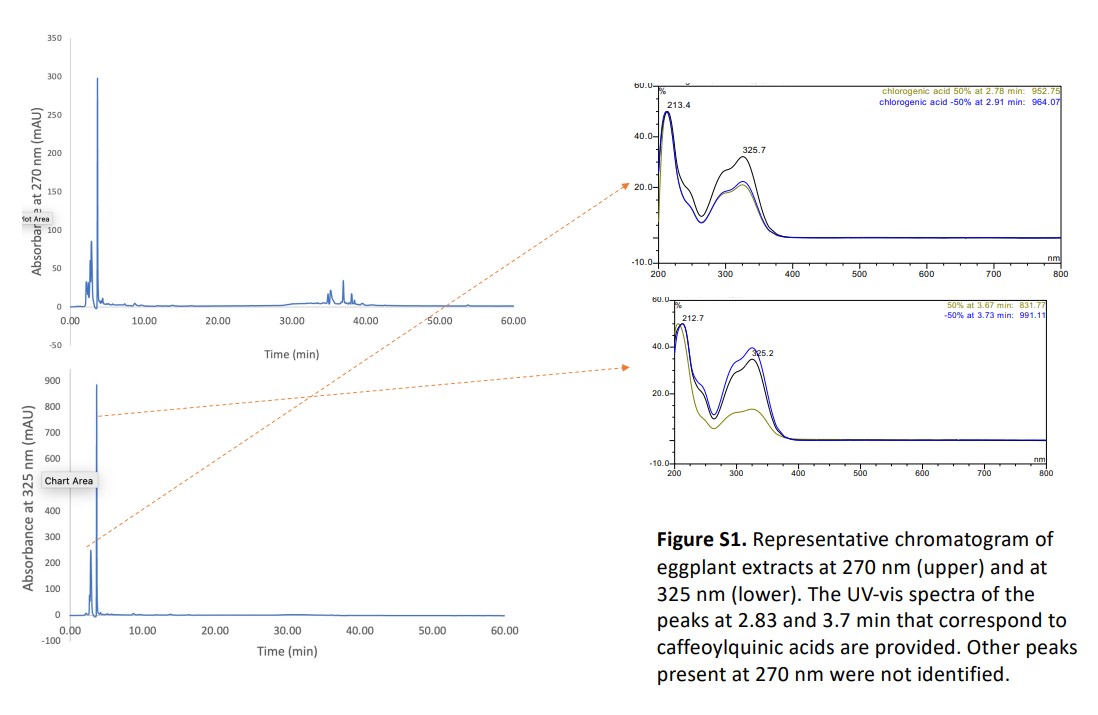

Supplement: Supplementary file 1 [file plants-11-02267-s001.zip › Figure S1.jpg]

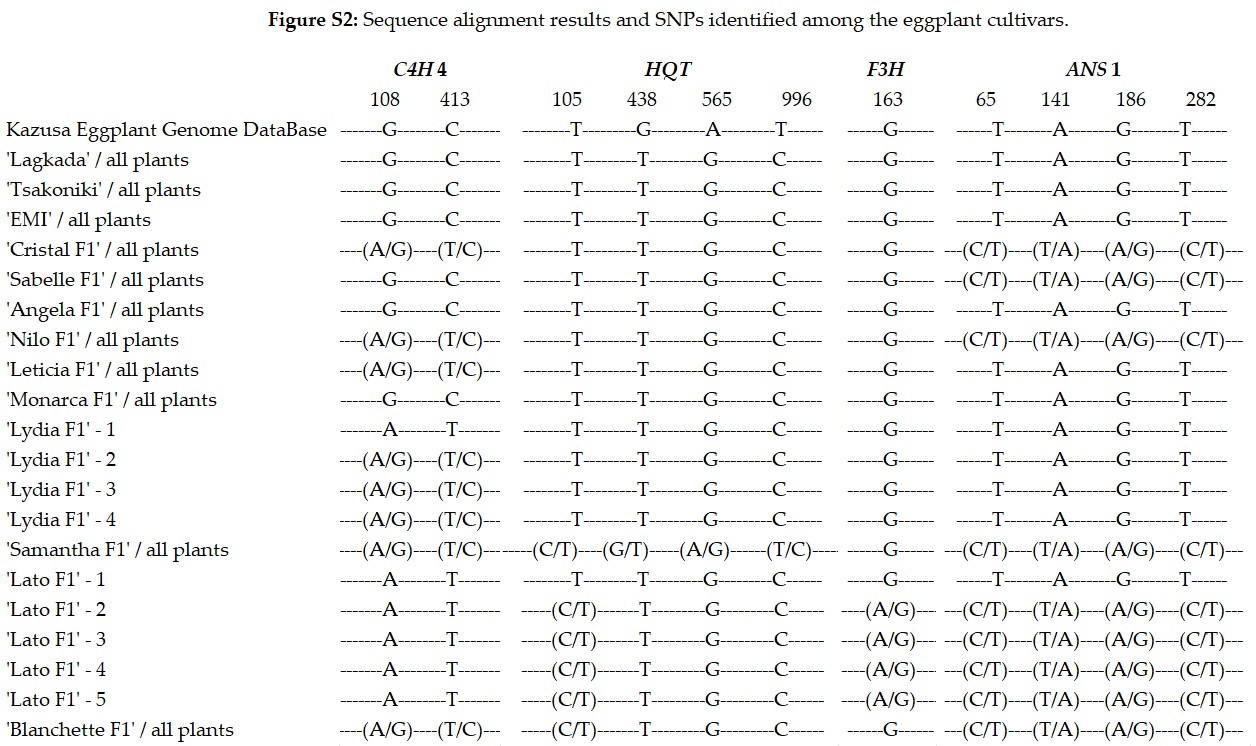

Supplement: Supplementary file 1 [file plants-11-02267-s001.zip › Figure S2.jpg]

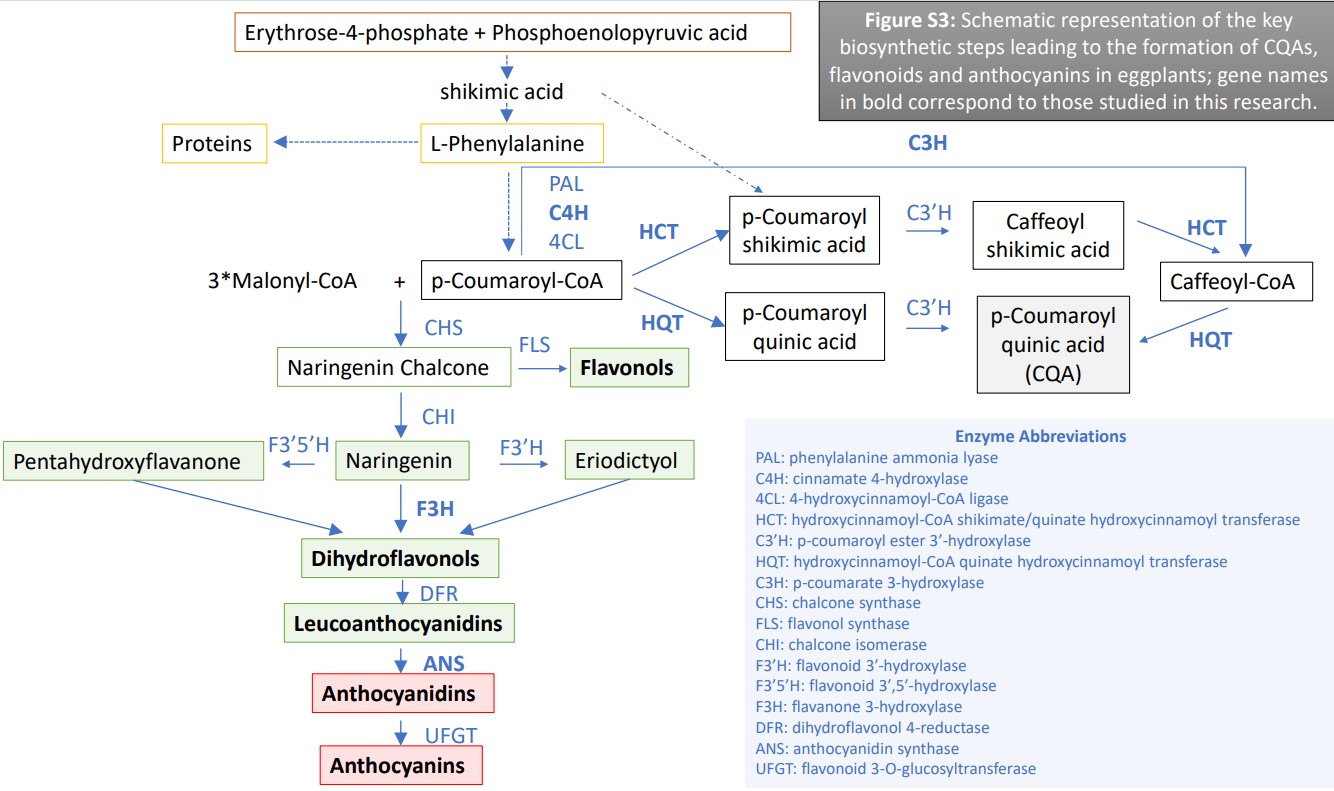

Supplement: Supplementary file 1 [file plants-11-02267-s001.zip › Figure S3.jpg]
